# Clinical outcomes and hospital-reported cost associated with surgical site infections and the co-occurrence of hospital-onset bacteremia and fungemia across US hospitals

**DOI:** 10.1017/ice.2025.13

**Published:** 2025-04

**Authors:** ChinEn Ai, Molly Jung, Samantha Bastow, Ghislene Adjaoute, David Bostick, Kalvin C. Yu

**Affiliations:** Department of Medical Affairs, Becton Dickinson and Company, Franklin Lakes, NJ, USA

## Abstract

**Objective:**

To evaluate the hospital-reported cost of care, clinical burden, and incidence of hospital-onset bacteremia and fungemia (HOB) for hospital admissions with surgical site infections (SSI).

**Methods:**

A cross-sectional study of 38 acute-care hospital admissions with a procedure under the National Healthcare Safety Network (NHSN) surveillance for SSI was conducted. SSI admissions were identified through NHSN reporting by the hospital. Clinical outcomes were estimated for SSI compared to no SSI controls using propensity matching and multivariable adjusted models that controlled for patient and hospital demographics; these endpoints were also compared for SSI admissions with and without HOB co-occurrence.

**Results:**

The rate of hospital-reported SSI was 0.15 per 100 admissions with a procedure under surveillance for SSI. Admissions with SSI compared to no SSI had significantly higher incremental hospital-reported cost of $30,689 and length of stay (LOS) was 11.6 days higher. The incidence of HOB was 6-fold higher in admissions with SSI compared to no SSI. For SSI admissions with HOB vs. no HOB, HOB added $28,049 to cost of care and 6.5 days to the LOS.

**Conclusions:**

Hospital-reported SSIs were associated with higher clinical and economic burden. Patients with SSI and HOB had even more deleterious outcomes. These data may inform programs to augment infection prevention bundles targeting SSIs and downstream complications or comorbidities like HOB.

## Introduction

Healthcare-associated infections (HAI) are associated with significant morbidity and mortality. Surgical site infections (SSI) affect between 1% and 20% of post-surgical patients and can be dependent on surgery type, age, obesity, level of immunosuppression, anatomical approach, and tissue condition.^1^ Modifiable risk factors include appropriate antibiotic prophylaxis, decolonization, and skin preparation.^1,2^ The National Healthcare Safety Network (NHSN) SSI guidelines require mandatory reporting of certain SSIs, and a subset can contribute to the Centers for Medicare and Medicaid Services (CMS) reimbursement, given the association with poor outcomes and the importance of peri-surgical strategies for infection risk mitigation.^3,4^

Surgery requires breaking the skin barrier and/or organ integrity which makes bloodstream infections (BSI) a particular concern in postoperative patients; also, SSIs may evolve into a BSI without timely and definitive intervention.^5,6^ Collectively, SSIs and BSIs (catheter and line-associated) account for up to 38% of HAI diagnoses.^7^

Recently, hospital-onset bacteremia and fungemia (HOB) has been proposed by the Centers for Disease Control and Prevention as a new CMS quality metric to include hospital-acquired BSIs from a more comprehensive list of sources, beyond what is currently reported.^8^ Therefore, studies that characterize the associated burden of HOB with currently reportable SSIs may inform quality improvement approaches in this vulnerable patient demographic. In this analysis, we estimate the attributable burden of SSIs and evaluate the incidence and clinical characteristics of HOB in SSI admissions within the NHSN reportable procedures and subsequent SSI surveillance period.

## Methods

### Study design and population

A cross-sectional propensity-matched analysis was conducted using data from 38 acute-care hospitals within the Becton Dickinson and Company (BD) Insights Database with adult inpatients aged ≥ 18 years and hospital length of stay (LOS) no greater than 365 days who were admitted between October 2015 and June 2019. The data analyzed included pharmacy, laboratory, administrative, patient demographic, admission, discharge, and transfer data. This analysis was reviewed and approved as a limited epidemiologic study and was granted an exemption from consent by the New England Institutional Review Board/WCG Human Subjects Research Committee (Wellesley, MA).

### Surgical site infection identification

Hospital-reported SSIs were those reported directly to the NHSN by infection preventionists. The control group included inpatient admissions with an NHSN-identified procedure eligible for SSI surveillance with a 30-day or 90-day surveillance period for SSI following the procedure. The 39 NHSN-operative procedures^9^ were collapsed into 9 mutually exclusive procedure categories to enable subgroup analyses: abdominal; cardiothoracic; colorectal; neurological; OB/GYN; orthopedic; transplant; vascular; and other (which includes breast surgery, neck surgery, and thyroid surgery). Details about the procedures that make up each subgroup are provided in Supplemental Table 1. The NHSN-reported SSI cases that were marked as “PATOS” (present at time of surgery), were excluded from the attributable burden of SSI and HOB evaluations.

A secondary analysis was performed for SSI cases identified through ICD-10-CM (the International Classification of Diseases, Tenth Revision, Clinical Modification) coding which was available for 80 hospitals, including the 38 hospitals in the primary analysis. An SSI admission was defined as a patient admission with ICD codes for infection following a procedure (T81.4) or postprocedural septic shock (T81.12XA) during the respective procedure surveillance period. For patients with multiple admissions with ICD-coded SSI during the NHSN procedure surveillance period for SSI, the first admission was used in the analysis. The data from the secondary analysis is provided in supplemental materials.

### Bloodstream infection endpoints

HOB was defined as the first non-duplicated positive blood culture collected within the hospital-onset period (on or after day 4 of hospitalization^10,11^); and community-onset bacteremia (COB) period was between one day prior to admission start and day 3 of admission^11^ for an eligible BSI organism as defined by the NHSN bloodstream pathogen list. COB was included from SSI readmissions after the procedure admission, during the respective NHSN procedure surveillance period. As the HOB relative to a SSI-event was of interest, the “at risk” period for HOB was at the same admission as the SSI or a subsequent readmission within the procedure surveillance period. In other words, an HOB event that occurred during an admission prior to the SSI-designated admission was not analyzed in this study. A subset of HOB was identified as potential BSI secondary to a SSI when the blood culture was collected during the NHSN SSI Module’s “17-day secondary BSI attribution period” (between 3 days prior to and 13 days after the positive SSI specimens^9^), and at least one organism matched in both blood and SSI specimen. As blood pathogens can be secondary to multiple sources, positive blood cultures that had pathogens that were also found in a respiratory or urine culture within 4 days prior and 2 days after the blood culture collection date were excluded.

### Outcome variables and other variables

Outcomes variables (LOS, hospital-reported cost, in-hospital mortality, and 30-day readmission), patient characteristics (sex, age, ever intensive care unit (ICU) status, diagnosis-related group, Acute Laboratory Risk of Mortality Score (ALaRMs) (an EHR-derived comorbidity measure that quantifies risk of inpatient death),^12–15^ insurance payor, and hospital demographics (staffed bed size, teaching status, and urbanicity) were obtained from the hospital administrative data. The three types of admissions that were used in the analysis were:

1. Procedure admission: The admission where the patient had the surgical procedure.
2. SSI admission: The first admission with a documented SSI. This could be a procedure admission in which a patient developed a SSI or a subsequent readmission following the procedure (within the SSI surveillance period).
3. HOB admission: The first admission with HOB occurring at a SSI admission or a subsequent readmission within the SSI surveillance period.

Hospital-reported costs are the expenses compiled and provided by the hospital healthcare system, representing the cost of care provided to a patient during their admission from the health system’s perspective.

### Statistical analysis

The rate and 95% confidence interval (CI) of SSI were calculated per 100 admissions with a NHSN-operative procedure under surveillance for SSI by the previously described 9 categories of procedure types using Poisson regression.

The attributable burden of SSI was estimated in a propensity-matched analysis comparing patients with SSI matched to patients without SSI (controls) excluding PATOS cases. The propensity scores were estimated using a logistic regression model with SSI status as the dependent variable with the following independent variables: NHSN-operative procedures, age group, sex, ALaRMS score, insurance payor, and hospital-level variables including staffed bed size, teaching status, and urbanicity. Propensity score matching was conducted using a 1:4 nearest match without replacement and 0.1 caliper. The distribution of patient, clinical, and hospital characteristics by SSI status were described using frequencies for categorical variables and means with standard deviations for continuous variables before and after propensity score matching. Differences in the distributions were estimated using χ^2^ tests and *t*-tests.

The attributable burden of SSI was estimated in the propensity-matched cohort using least mean estimation from multivariable adjusted generalized mixed models with hospitals as a random effect to account for within-cluster correlation. Poisson regression was used for LOS, gamma regression for total cost, and binominal regression for binary outcomes (30-day readmission, in-hospital mortality, and risk of HOB). COB was not included in the multivariable-adjusted analyses due to low prevalence (*P* < 0.5). Models were adjusted for age, sex, ALaRMS score, insurance payor, and hospital-level variables including staffed bed size, teaching status, and urbanicity.

The incremental burden of SSI with HOB (cases were HOB admissions) was quantified compared to those with SSI without HOB (controls were SSI admissions without HOB) using the abovementioned multivariable-adjusted models. Due to the limited sample size of patients with SSI (with and without HOB), a propensity score-matched analysis was not performed.

Hospital-reported SSI data included the event reported date and pathogen which allowed for a pathogen-level analysis. To give visibility to the pathogens associated with the infection types, the distribution of pathogen^16^ (including commensal pathogens) in positive SSI source culture and positive blood culture meeting the NHSN criteria for potential secondary BSI were quantified using frequencies by 30-day or 90-day SSI surveillance periods (for respective procedure type). PATOS cases were included to provide comprehensive insights. BSIs that were potentially secondary to SSIs were identified when the difference in days was within the NHSN SSI Module’s 17-day secondary BSI attribution window within the same admission, and when the same pathogen was present in SSI and HOB blood culture specimens.^9^ The difference in days between the SSI event date and HOB were estimated to quantify the timing between SSI and HOB occurrence.

All analyses were conducted using R software version 4.1.2.^17^

## Results

### Demographics

The distribution of patient, clinical, and hospital characteristics before and after propensity score matching is shown in Table 1. Prior to the match, there were differences in the distribution of most variables by SSI status. Patients with SSI compared to no SSI tended to be older (18–40 years of age category was 9.7% vs 26.3% for case and control, respectively), male (43.5% vs 37.2%, respectively), and have higher ALaRM Scores (42.7 vs 33.6, respectively), and came from moderate sized facilities. After propensity score matching, the distributions were no longer statistically different, and the standardized mean differences were generally *P* < 0.1 (Supplemental Table 2) suggesting improved comparability after propensity score matching. Notably, ever ICU utilization was higher in admissions with SSI than without SSI before and after propensity score matching (53.7% vs 19.8% and 53.5% vs 31.8%, respectively).

**Table 1.** Patient characteristics for hospital-reported SSI (excluding PATOS) and NHSN procedure surveillance period before and after propensity score matching, October 2015 through June 2019

Characteristics	Before propensity score matching			After propensity score matching		
	SSI	No SSI (Control)	*P*	SSI	No SSI (Control)	*P*
	(N = 216)	(N = 145999)		(N = 215)	(N = 858)	
Age_group			<0.0001			0.7
18–40	21 (9.7%)	38372 (26.3%)		21 (9.8%)	70 (8.2%)	
41–64	109 (50.5%)	51112 (35.0%)		108 (50.2%)	413 (48.1%)	
65–80	73 (33.8%)	44737 (30.6%)		73 (34.0%)	317 (36.9%)	
>80	13 (6.0%)	11778 (8.1%)		13 (6.0%)	58 (6.8%)	
Male	94 (43.5%)	54293 (37.2%)	<0.0001	93 (43.3%)	346 (40.3%)	0.5
Ever ICU	116 (53.7%)	28879 (19.8%)	<0.0001	115 (53.5%)	273 (31.8%)	<0.0001
ALaRM Score, mean (SD)	42.7 (19.0)	33.6 (18.6)	<0.0001	42.4 (18.6)	41.9 (18.9)	0.7
Payor			<0.001			0.8
Medicaid	22 (10.2%)	16091 (11.0%)		22 (10.2%)	76 (8.9%)	
Medicare	110 (50.9%)	63583 (43.6%)		109 (50.7%)	467 (54.4%)	
Other	6 (2.8%)	4852 (3.3%)		6 (2.8%)	28 (3.3%)	
Private	72 (33.3%)	56985 (39.0%)		72 (33.5%)	261 (30.4%)	
Uninsured	6 (2.8%)	4488 (3.1%)		6 (2.8%)	26 (3.0%)	
Facility bed size			<0.0001			0.8
<100	18 (8.3%)	5161 (3.5%)		17 (7.9%)	71 (8.3%)	
100–300	89 (41.2%)	36550 (25.0%)		89 (41.4%)	331 (38.6%)	
>300	109 (50.5%)	104288 (71.4%)		109 (50.7%)	456 (53.1%)	
Teaching Facility	108 (50.0%)	96258 (65.9%)	<0.001	108 (50.2%)	431 (50.2%)	>0.9
Urban Facility	177 (81.9%)	120117 (82.3%)	0.7	176 (81.9%)	739 (86.1%)	0.1

Abbreviations: SSI, surgical site infection; NHSN, National Healthcare Safety Network; PATOS, [infection] present at time of surgery; ICU, intensive care unit; ALaRM Score, acute laboratory risk of mortality score; SD, standard deviation.

Note. *P* values were estimated using χ^2^ tests and Student’s *T*-tests.

**Table 2.** Hospital-reported SSI rate (excluding PATOS) per 100 admissions by procedure category

Procedure Category	Number of SSI /Number of admissions with procedure	Unadjusted rate (95%CI)
Overall	216/146,204	0.15 (0.13, 0.17)
Abdominal	20/31,285	0.06 (0.04, 0.10)
Cardiothoracic	6/14,202	0.04 (0.02, 0.09)
Colorectal	145/15,216	0.95 (0.81, 1.12)
Neurological	0/4,332	0
Obstetrics and Gynecology	12/31,212	0.04 (0.02, 0.06)
Orthopedic	27/52,102	0.05 (0.04, 0.07)
Other	2/3,103	0.06 (0.01, 0.20)
Transplant	0/1,311	0
Vascular	4/4,091	0.10 (0.03, 0.23)

Abbreviations: SSI, surgical site infection; PATOS, [infection] present at time of surgery; CI, confidence interval.

Note. The surgery categories were based on the 39 NHSN-operative procedures types eligible for SSI surveillance and collapsed to the follow 9 mutually exclusive groups: **abdominal** (appendix surgery, bile duct, liver or pancreatic surgery, gallbladder surgery, gastric surgery, herniorrhaphy, kidney surgery, prostate surgery, spleen surgery, and exploratory laparotomy); **cardiothoracic** (cardiac surgery, coronary bypass with chest & donor incisions, coronary bypass graft with chest incision, pacemaker surgery, and thoracic surgery); **colorectal** (colon surgery, rectal surgery and small bowel surgery); **neurological** (craniotomy and ventricular shunt); **obstetrics and gynecology** (cesarean section, abdominal hysterectomy, ovarian surgery, and vaginal hysterectomy); **orthopedic** (limb amputation, spinal fusion, open reduction of fracture, hip prosthesis, knee prosthesis, and laminectomy); **other** (breast surgery, neck surgery, and thyroid and/or parathyroid surgery); **transplant** (heart transplant, kidney transplant, and liver transplant); and **vascular** (abdominal aortic aneurysm repair, arteriovenous shunt for dialysis, carotid endarterectomy, peripheral vascular bypass surgery).

### Rate of SSI

Among 38 hospitals, there were 146,215 admissions with an NHSN reportable procedure of which 146,204 had a documented procedure and the SSI rate was estimated. The overall rate of hospital-reported SSI was 0.15 per 100 admissions with procedure (Table 2). The procedures with the highest hospital-reported SSI incidence per 100 admissions were colorectal (0.95), vascular (0.10), abdominal (0.06), and other (0.06) (Table 2). Approximately 31% (n = 67) of the hospital-reported SSI events also had an ICD-coded SSI (data not shown). The secondary analysis using ICD-coded data demonstrated an SSI rate of 1.49 per 100 admissions (Supplemental Table 3).

### Burden of SSI

Of 216 hospital-reported SSI cases, one case did not match and was excluded in subsequent analysis. The adjusted incremental burden of hospital-reported SSI, except risk of 30-day readmission and COB, was significantly higher across all other outcomes: hospital-reported cost of care ($30,689), LOS (11.6 days), relative risk (RR) of in-hospital mortality (RR = 3.4) and risk of HOB (RR = 6.1) (Table 3).

**Table 3.** Model estimated financial and clinical outcomes by hospital-reported SSI compared to SSI-free controls with procedures under NHSN surveillance for SSI in a propensity score-matched analysis (excluding PATOS), October 2015 through June 2019

	Admissions with SSI	Admissions without SSI	Estimated Difference/RR	*P*
	(N = 215)	(N = 858)	(95% CI)	
Financial outcomes				
Total cost, $ (95% CI)	$53,104 ($42,891, $65,749)	$22,415 ($18,357, $27,371)	$30,689 ($29,900, $31,477)	<0.0001
LOS, days (95% CI)	18.6 (16.6, 20.9)	7.0 (6.3, 7.9)	11.6 (11.4, 11.7)	<0.0001
Clinical outcomes				
30-day Readmission, % (95% CI)	13.4 (7.6, 22.3)	9.6 (5.8, 15.5)	1.5 (0.9, 2.3)	0.1
In-hospital mortality, % (95% CI)	0.2 (0, 100)	0.06 (0, 100)	3.4 (1.7, 7.0)	0.0008
HOB, % (95% CI)	9.3 (4.0, 19.8)	1.7 (0.7, 3.9)	6.1 (3.2, 11.5)	<0.0001

Abbreviations: SSI, surgical site infection; NHSN, National Healthcare Safety Network; PATOS, [infection] present at time of surgery; RR, relative risk; CI, confidence interval; LOS, length of stay; HOB, hospital-onset bacteremia; COB, community-onset bacteremia.

Models were adjusted for age, sex, ALaRMS score, insurance payer type, and hospital-level variables (staffed bed size, teaching status, and urbanicity).

### SSI and HOB outcomes

Of the 216 hospital-reported SSI cases, 2 (0.9%) had COB and 27 (12.5%) had HOB (data not shown). In a subgroup analysis of patients with SSI, HOB co-occurrence was associated with a statistically significantly higher cost of care ($28,049; Table 4). In addition, admissions with SSI and HOB co-occurrence were associated with a longer LOS (6.5 days) than those with SSI and no HOB (*P* < 0.0001) and 3.5-fold higher 30-day readmission (*P =* 0.04). HOB in the setting of hospital-reported SSI was associated with higher mortality (3.0-fold higher); however, the difference did not reach statistical significance.

**Table 4.** Model estimated financial and clinical outcomes by hospital-reported SSI with HOB compared to SSI without HOB (excluding PATOS), October 2015 through June 2019

	SSI admission with HOB	SSI admission without HOB	Estimated Difference/RR	*P*
	(N = 27)	(N = 189)	(95% CI)	
Financial outcomes				
Total cost, $ (95% CI)	$65,857 ($42,428, $102,223)	$37,807 ($26,606, $53,724)	$28,049 ($22,394, $33,705)	0.0004
LOS, days (95% CI)	21.2 (17.8, 25.1)	14.6 (12.5, 17.1)	6.5 (5.8, 7.2)	<0.0001
Clinical outcomes				
30-day Readmission, % (95% CI)	0.6 (0, 100)	0.2 (0, 100)	3.5 (1.1, 11.7)	0.04
In-hospital mortality, % (95% CI)	0 (0, 100)	0 (0, 100)	3.0 (0.8, 11.5)	0.1

Note. The analysis did not implement a propensity-match cohort due to limited sample size.

Abbreviations: SSI, surgical site infection; HOB, hospital-onset bacteremia; PATOS, [infection] present at time of surgery; RR, relative risk; CI, confidence interval; LOS, length of stay; COB, community-onset bacteremia.

Models were adjusted for age, sex, ALaRMS score, insurance payer type, and hospital-level variables (staffed bed size, teaching status, and urbanicity).

### SSI and HOB relative timing

**Figure 1.**
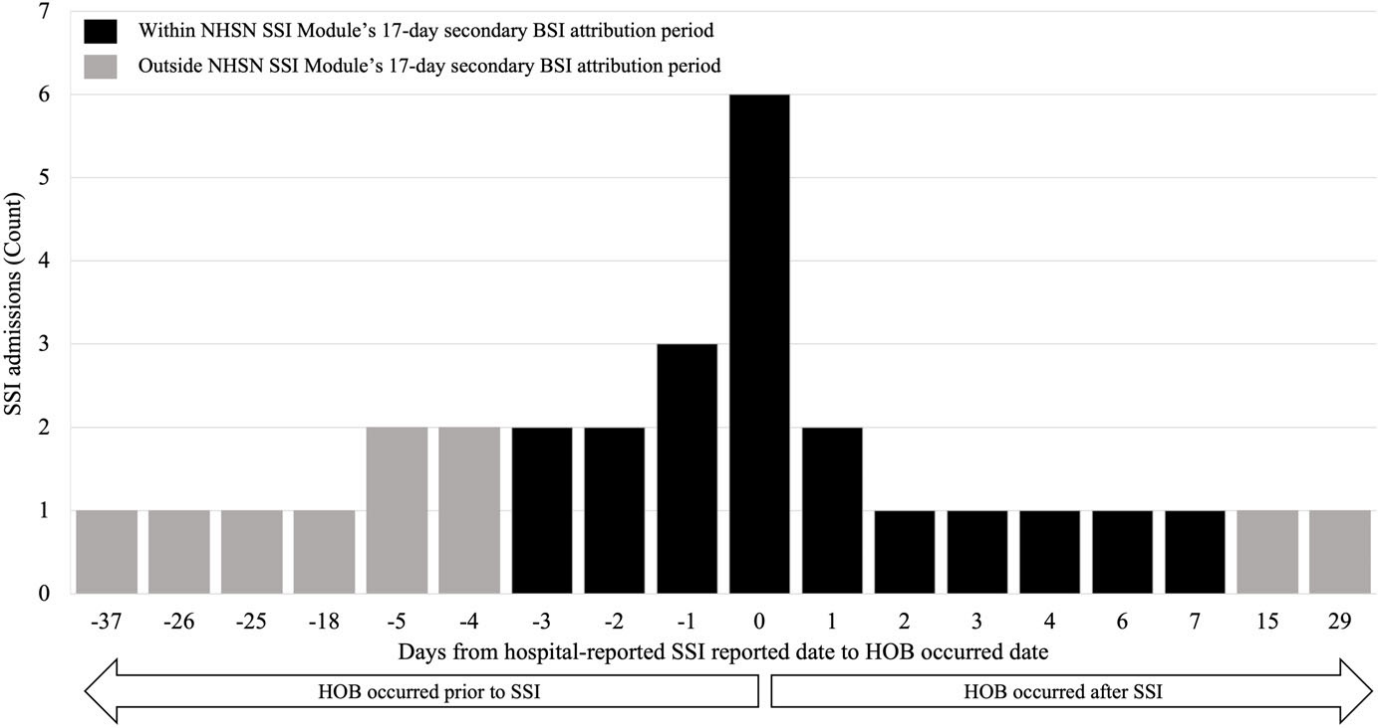
Days difference from hospital-reported surgical site infection (SSI) reported date to SSI-associated HOB occurred date and secondary BSI attribution period, hospital-reported SSI cohort. *Note*: Days from hospital-reported SSI reported date to HOB occurred date: day difference between date of SSI reported to National Healthcare Safety Network (NHSN) and HOB blood specimen collection date; Secondary BSI attribution period: NHSN SSI Module’s 17-day secondary BSI attribution period, 3 days prior and 13 days after date of hospital-reported SSI event.

In addition to the 27 HOB SSI co-occurrent cases, three additional PATOS cases were included in this analysis for a total of 30 HOB SSI admissions. Approximately 33% (n = 10/30) of hospital-reported SSI with HOB met the NHSN criteria for secondary BSI. Of the 30 admissions with SSI and HOB co-occurrence, 20 occurred within the 17-day attribution period (Figure 1); of these 20 admissions, 10 HOB events had at least one organism matching an organism identified from the SSI event-related specimens (data not shown).

### Pathogen distribution

All 242 NSHN hospital-reported SSI cases (including PATOS) were evaluated for the pathogen prevalence of positive SSI source culture and potential BSI secondary to SSI (HOB or commensal events) by 30-day or 90-day surveillance period (Table 5); the rows of this table are not additive since patients could have multiple procedures and multiple types of pathogens. The prevalent pathogens in all hospital-reported SSI differed by the surveillance period (30-day vs. 90-day). Of the 185 hospital-reported SSI admissions with a 30-day surveillance, Enterobacteriaceae was the most common microorganism (46.5%) followed by Enterococcus spp (42.7%). Nine admissions (30-day surveillance) had BSI potentially secondary to SSI, with the most common BSI-SSI matched pathogen being Enterococcus spp. (55.6%) followed by other Candida species (22.2%).

**Table 5.** Microorganisms in positive SSI source cultures and potential BSI secondary to SSI (including PATOS) stratified by SSI surveillance period, October 2015 through June 2019

	Positive SSI source culture N (%)	Positive blood culture meeting NHSN criteria for secondary BSI (ie, matched pathogen) N (%)
**30-day NHSN surveillance period for SSI**		
SSI with SSI source culture	185	9
Candida albicans and C. auris	27 (14.6%)	0 (0%)
CoNS	14 (7.6%)	0 (0%)
Enterobacteriaceae	86 (46.5%)	1 (11.1%)
Enterococcus spp.	79 (42.7%)	5 (55.6%)
Environmental GNB	4 (2.2%)	0 (0%)
Pseudomonas aeruginosa	17 (9.2%)	0 (0%)
Other Candida spp.	16 (8.6%)	2 (22.2%)
Other commensal	29 (15.7%)	1 (11.1%)
Other GNB	52 (28.1%)	1 (11.1%)
Other GPB	30 (16.2%)	0 (0%)
S. aureus	14 (7.6%)	0 (0%)
No specimen	15	NA
**90-day NHSN surveillance period for SSI**		
SSI with SSI source culture	41	6
Candida albicans and C. auris	0 (0%)	0 (0%)
CoNS	12 (29.3%)	1 (16.7%)
Enterobacteriaceae	8 (19.5%)	0 (0%)
Enterococcus spp.	5 (12.2%)	0 (0%)
Environmental GNB	2 (4.9%)	1 (16.7%)
Pseudomonas aeruginosa	3 (7.3%)	0 (0%)
Candida spp.	2 (4.9%)	0 (0%)
Other commensal	7 (17.1%)	1 (16.7%)
Other GNB	2 (4.9%)	0 (0%)
Other GPB	3 (7.3%)	0 (0%)
S. aureus	19 (46.3%)	3 (50.0%)
No specimen	1	NA

Abbreviations: SSI, surgical site infection; BSI, bloodstream infection; HOB, hospital-onset bacteremia; PATOS, [infection] present at time of surgery; CoNS, coagulase-negative staphylococci; GNB, gram-negative bacteria; GPB, gram-positive bacteria.

Note. “Potential BSI secondary to SSI” = matched pathogen from SSI source culture within the secondary BSI 17-day attribution period; environmental GNB includes Acinetobacter species, Pseudomonas species (excluding Pseudomonas aeruginosa), and Serratia species.

The two most common pathogens in the 41 SSI admissions with a procedure with a 90-day surveillance were S. aureus (46.3%) and coagulase-negative staphylococci (CoNS) (29.3%). In the 6 admissions (90-day surveillance) with potential secondary BSI to SSI, S. aureus (50.0%) was the most common SSI and BSI matched pathogen in this group. One hospital-reported SSI admission had a readmission with community-onset Bacteroides fragilis bacteremia within the NHSN defined secondary attribution period (data not shown).

## Discussion

SSIs can progress systemically and healthcare-associated BSIs are associated with a 3–4-fold increase in mortality, prolonged hospitalization, and increased costs.^18–20^ Given that HOB is planned by the NHSN for volunteer reporting, an epidemiologic evaluation of SSI-associated BSIs was conducted with the delineations of HOB and COB to better understand the burden in a way that may inform HOB mitigation programs.

A SSI rate of 0.15 per 100 admissions was observed when identified by hospital-reporting-to-NHSN. In other studies, ICD-coded data for SSI identification has been used to estimate SSI burden; therefore, a secondary ICD coding-based SSI analysis was performed and resulted in an SSI rate of 1.49 per 100 admissions and a higher associated risk with HOB co-occurrence (Supplemental Tables 3 and 5). Overestimation of SSI rates using ICD-coding methodology has been previously described and may be a result of misclassification or lack of specificity and granularity; for example, PATOS cases are likely captured in this methodology.^21^ Interestingly, HOB co-occurrence was higher (vs controls) for both methods of identifying SSIs, suggesting higher risk in SSI admissions overall. The adjusted incremental burden of hospital-reported SSI was significantly higher compared to procedure admissions without SSI (control) for incremental hospital-reported cost, added LOS, RR of in-hospital mortality, and RR of 30-days readmission. These contemporary findings update previous publications where similar SSI outcomes were reported but without the context of associated HOB.^22–25^

The relative risk of HOB co-occurrence with SSI, whether derived from either hospital-reported or ICD methodologies, was approximately 6-fold higher compared to controls, with an overall HOB incidence of 12.5% for hospital-reported SSI admissions. HOB co-occurrence in SSI admissions was associated with a significantly higher cost of care and hospital LOS compared to control (SSI admission without HOB). These results augment a 2002 SSI and postoperative BSI surveillance evaluation of 40,191 surgeries which found a SSI incidence rate of 1.3% (n = 515) and postoperative bacteremia secondary to SSI rate of 9.1% (n = 47).^26^ A more recent study reported that non-CLABSI HOB was associated with an incremental LOS of 12.1–14.9 days, incremental cost of care $25,207–$42,095, and RR of 30-day readmission of 1.06–1.45; thus, this analysis is unique in highlighting the intersection of SSI and HOB co-occurrence outcomes.^11^

Approximately two-thirds of HOB cases did not meet the NSHN criteria for secondary BSI from SSI. This could be due to several factors including: not all SSIs have a feasible source available to culture (ie, anatomical incision cellulitis); even if cultures are performed quality of the specimen and mixed flora growth can be a confounding phenomenon. Therefore, while some instances of HOB may be attributable to SSI, in many other cases, HOB and SSI may coexist as comorbidities. Consequently, their overlapping risk factors warrant further investigation and consideration within infection prevention programs. More specifically, visibility to the BSI pathogen can sometimes inform source control policies or aid in root cause analyses. One readmission SSI case of COB with Bacteroides fragilis implicates a gastrointestinal procedure as the likely source. Thus, while COB is a designation meant to convey “community onset” bacteremia events, with SSIs it is very possible a COB event in post-surgical readmissions can reflect important healthcare-associated complications. Cross referencing pathogen source with procedures can also be done at a higher level for root cause analyses. For example, differences in the distribution of top pathogens in a SSI culture was notable for increased S. aureus (known skin source) and related matched HOB bacteremia cases in 90-day surveillance procedures.

Limitations of this study should be acknowledged. The burden of SSI was quantified using data from the first SSI during the surveillance period; as a result our estimate is a conservative estimate and may not represent the total SSI burden. The analysis was conducted on a convenience sample of admissions within the acute care setting and does not encompass procedures at ambulatory surgery centers or outpatient facilities, who may be considered lower-risk admissions. Therefore, the study population may overestimate the incidence of SSI relative to the general surgical population. Readmission was only available if patients went to the same hospital; this may lead to underestimating the burden if patients sought post-surgical care at another facility. Conversely, overestimation of SSI could be a factor with the inclusion of cases with infection present at time of surgery (PATOS). For this reason, PATOS cases were not included in the attributable burden of SSI and HOB. While PATOS cases do not currently contribute to the CMS SSI standardized infection ratio and related penalties, they may still serve as a source of secondary HOB so they were included in the pathogen-level analysis; as a principal aim of this study was to better quantify the overall burden and relationship of HOB co-occurrence with SSI admissions, the inclusion of PATOS is important as it reflects clinical reality in real world data and the practice of medicine. The pathogen-level analysis focused on the first SSI event although it is possible for multiple SSIs to occur; however, this was likely not an issue as only 2% (n = 5/242) of admissions with hospital-reported SSI had multiple SSIs in this study sample. Lastly, hospital-reported cost of care was used and the calculation method may vary between institutions.

It is worth noting that the analysis was not designed to prove SSI and HOB causality (with the possible exception of the matched surgical wound and blood culture organism subset); rather, it delineated the epidemiological associations and burden of these events in SSI admissions to inform targeted infection prevention and antimicrobial stewardship programs on associated BSI events. Other considerations regarding HOB as a metric likely need further investigation, such as applicability of special populations like immune-compromised surgical patients where HOB may not be preventable.^27^ Similarly unanswered are insights on PATOS events as a potential source of HOB. Thus, this analysis may incite further studies and potentially improve programs aimed at SSI source control for subsequent BSI events.

## Conclusion

SSI admissions were associated with significant increases in clinical and economic burden. When compared to controls, SSI admissions with associated HOB had additional incremental burden in cost of care and LOS. Depending on when a SSI develops, SSI patients can pose an epidemiological demographic risk for HOB and COB BSI events. These findings highlight the importance of upstream SSI prevention bundles and vigilant attention to BSI risk-factors to help mitigate increased cumulative morbidity associated with the care of SSI patients.

## Supporting information

Ai et al. supplementary material Ai et al. supplementary material

## Supplementary material

The supplementary material for this article can be found at https://doi.org/10.1017/ice.2025.13
