# Supplementary material for: Clinical outcomes and hospital-reported cost associated with surgical site infections and the co-occurrence of hospital-onset bacteremia and fungemia across US hospitals
Source: Infect Control Hosp Epidemiol. 2025 Feb 19;46(4):391–7. doi: 10.1017/ice.2025.13 (PMC12015625; doi:10.1017/ice.2025.13)
Supplement: Ai et al. supplementary material [file S0899823X25000133sup001.docx]

**Supplemental Table 1. NHSN operative procedures and respective procedure category.**

| **Procedure category** | **NHSN operative procedure** |
| --- | --- |
| **30-day surveillance** | |
| Abdominal | Appendix surgery (APPY) |
| Abdominal | Bile duct, liver or pancreatic surgery (BILI) |
| Abdominal | Exploratory laparotomy (XLAP) |
| Abdominal | Gallbladder surgery (CHOL) |
| Abdominal | Gastric surgery (GAST) |
| Abdominal | Kidney surgery (NEPH) |
| Abdominal | Prostate surgery (PRST) |
| Abdominal | Spleen surgery (SPLE) |
| Cardiothoracic | Thoracic surgery (THOR) |
| Colorectal | Colon surgery (COLO) |
| Colorectal | Rectal surgery (REC) |
| Colorectal | Small bowel surgery (SB) |
| Other | Neck surgery (NECK) |
| Other | Thyroid and/or parathyroid surgery (THYR) |
| Obstetrics and Gynecology | Abdominal hysterectomy (HYST) |
| Obstetrics and Gynecology | Cesarean section (CSEC) |
| Obstetrics and Gynecology | Ovarian surgery (OVRY) |
| Obstetrics and Gynecology | Vaginal hysterectomy (VHYS) |
| Orthopedic | Laminectomy (LAM) |
| Orthopedic | Limb amputation (AMP) |
| Transplant | Heart transplant (HTP) |
| Transplant | Kidney transplant (KTP) |
| Transplant | Liver transplant (LTP) |
| Vascular | Abdominal aortic aneurysm repair (AAA) |
| Vascular | AV shunt for dialysis (AVSD) |
| Vascular | Carotid endarterectomy (CEA) |
| **90-day Surveillance** | |
| Abdominal | Herniorrhaphy (HER) |
| Cardiothoracic | Cardiac surgery (CARD) |
| Cardiothoracic | Coronary bypass graft with chest incision (CBGC) |
| Cardiothoracic | Coronary bypass with chest & donor incisions (CBGB) |
| Cardiothoracic | Pacemaker surgery (PACE) |
| Other | Breast surgery (BRST) |
| Neurological | Craniotomy (CRAN) |
| Neurological | Ventricular shunt (VSHN) |
| Orthopedic | Hip prosthesis (HPRO) |
| Orthopedic | Knee prosthesis (KPRO) |
| Orthopedic | Open reduction of fracture (FX) |
| Orthopedic | Spinal fusion (FUSN) |
| Vascular | Peripheral vascular bypass surgery (PVBY) |

**Supplemental Table 2. Standardized mean differences before and after propensity matching adjustment.**

|  | **Standardized Mean Difference** | |
| --- | --- | --- |
|  | Before | After |
| Age group, 18-40 | 0.9581 | 0.0549 |
| Age group, 41-64 | 0.4494 | 0.0419 |
| Age group, 65-80 | 0.1547 | -0.0639 |
| Age group, >80 | 0.0589 | -0.0293 |
| Female | 0.2999 | -0.0586 |
| ALaRM Score | 0.6447 | 0.0205 |
| Payor, Medicaid | 0.1406 | 0.0423 |
| Payor, Medicare | 0.2913 | -0.0744 |
| Payor, Other | 0.0062 | -0.0283 |
| Payor, Private | 0.2119 | 0.0666 |
| Payor, uninsured | 0.0136 | -0.0142 |
| Facility size, <100 | 0.1500 | -0.0168 |
| Facility size, 100-<300 | 0.2860 | 0.0567 |
| Facility size, ≥ 300 | 0.3644 | -0.0465 |
| Teaching facility | 0.2244 | 0.0023 |
| Urban facility | 0.0342 | -0.1119 |
| Procedure, AAA | 0.0000 | 0.0490 |
| Procedure, BILI | 0.0218 | 0.1126 |
| Procedure, BRST | 0.0342 | 0.0422 |
| Procedure, CBGB | 0.0363 | 0.1593 |
| Procedure, CHOL | -0.0456 | 0.9566 |
| Procedure, COLO | -0.0108 | 1.1228 |
| Procedure, CSEC | 0.0000 | 3.3804 |
| Procedure, FUSN | -0.0187 | 0.0057 |
| Procedure, FX | 0.0747 | 0.6540 |
| Procedure, GAST | 0.0123 | 0.1427 |
| Procedure, HER | 0.0000 | 0.0768 |
| Procedure, HPRO | -0.0504 | 0.1122 |
| Procedure, HYST | -0.0109 | 0.0362 |
| Procedure, KPRO | 0.0156 | 0.6651 |
| Procedure, NECK | 0.0322 | -0.0936 |
| Procedure, PACE | 0.0000 | -0.0393 |
| Procedure, PRST | -0.0161 | -0.0005 |
| Procedure, PVBY | 0.0000 | 0.0949 |
| Procedure, SB | -0.0104 | -0.0393 |
| Procedure, THOR | -0.0322 | 0.0126 |
| Procedure, VHYS | 0.0114 | 0.0580 |
| Procedure, XLAP | 0.0162 | 0.0740 |

**Methods for ICD-coded SSI analysis:**

**Definition of ICD-coded SSI**

ICD-coded SSI identification was available in 80 hospitals. A SSI admission was defined as a patient admission with ICD codes for infection following a procedure (T81.4) or postprocedural septic shock (T81.12XA) during the respective procedure surveillance period. For patients with multiple admissions with ICD-coded SSI during the NHSN procedure surveillance period for SSI, the first admission was used in the analysis.

**Statistical analysis**

The rate of SSI was calculated per 100 admissions with a NHSN procedure under surveillance for SSI by 9 categories of procedure types. To improve comparability between admissions with and without SSI, the non-SSI control group was restricted to patients who had the same procedures: exact same procedure code (88% of SSI cases) or same procedure category (12%). The distribution of patient, clinical, and hospital characteristics of patients with SSI and controls were described using frequencies for categorical variables and medians with interquartile ranges for continuous variables.

Least Squares Mean estimation of multivariable adjusted generalized mixed models were used with hospitals as a random effect to account for within-cluster correlation to estimate the incremental burden SSI. Data from the SSI admission (case) was compared to the procedure admission without SSI (control). Poisson regression for LOS, gamma regression for total cost, and binominal regression for in-hospital mortality, risk of HOB, risk of COB, and 30-day readmission rates were employed. Models were adjusted for age, sex, ALaRMS score, insurance payor, and hospital-level variables including staffed bed size, teaching status, and urbanicity.

To evaluate the incremental burden of HOB in SSI, a subgroup analysis restricted to patients with SSI was conducted. The incremental burden of SSI with HOB (cases were HOB admissions) was quantified compared to those with SSI without HOB (controls were SSI admissions without HOB) using the abovementioned multivariable adjusted models.

**Supplemental Table 3. ICD-coded SSI rate per 100 admissions by procedure category.**

| **Procedure Category** | **Number of ICD coded SSI/Number of admissions with procedure** | **ICD coded SSI rate (95% CI)** |
| --- | --- | --- |
| Overall | 3,312/222,298 | 1.49 (1.44, 1.54) |
| Abdominal | 1,304/47,282 | 2.76 (2.61, 2.91) |
| Cardiothoracic | 333/20,987 | 1.59 (1.42, 1.76) |
| Colorectal | 979/22,509 | 4.35 (4.08, 4.62) |
| Neurological | 148/6,755 | 2.19 (1.84, 2.54) |
| Obstetrics and Gynecology | 153/49,940 | 0.31 (0.26, 0.35) |
| Orthopedic | 932/78,773 | 1.18 (1.11, 1.26) |
| Other | 152/4,445 | 3.42 (2.88, 3.96) |
| Transplant | 47/1,736 | 2.71 (1.93, 3.48) |
| Vascular | 107/5,859 | 1.83 (1.48, 2.17) |

Note: The counts are not additive because some patients had multiple procedures so they could be represented in multiple procedure categories.

Abbreviations: ICD, international classification of diseases; SSI, surgical site infection; rate, SSI rate per 100 admissions with procedure; CI, confidence interval; n, number of SSI admissions; N, number of admissions with procedure. The surgery categories were based on the 39 NHSN-operative procedures types eligible for SSI surveillance and collapsed to the follow 9 mutually exclusive groups: abdominal (appendix surgery, bile duct, liver or pancreatic surgery, gallbladder surgery, gastric surgery, herniorrhaphy, kidney surgery, prostate surgery, spleen surgery, and exploratory laparotomy); cardiothoracic (cardiac surgery, coronary bypass with chest & donor incisions, coronary bypass graft with chest incision, pacemaker surgery, and thoracic surgery); colorectal (colon surgery, rectal surgery and small bowel surgery); neurological (craniotomy and ventricular shunt); obstetrics and gynecology (cesarean section, abdominal hysterectomy, ovarian surgery, and vaginal hysterectomy); orthopedic (limb amputation, spinal fusion, open reduction of fracture, hip prosthesis, knee prosthesis, and laminectomy); other (breast surgery, neck surgery, and thyroid and/or parathyroid surgery); transplant (heart transplant, kidney transplant, and liver transplant); and vascular (abdominal aortic aneurysm repair, arteriovenous shunt for dialysis, carotid endarterectomy, peripheral vascular bypass surgery).

**Supplemental Table 4. Distributions of patient, clinical, and facility characteristics of patients with a NHSN-identified procedure for SSI surveillance with and without ICD-coded SSI from 80 hospitals.**

|  | **SSI**  **(Case)** | **No SSI**  **(Control)** |
| --- | --- | --- |
| N | 3,312 | 208,925 |
| SSI Surveillance period^*^ |  |  |
| 30-day only | 2,185 (66.0%) | 125,791 (60.2%) |
| 90-day only | 1,515 (45.7%) | 95,221 (45.6%) |
| Age group |  |  |
| 18-40 | 391 (11.8%) | 59,194 (28.3%) |
| 41-64 | 1,582 (47.8%) | 70,866 (33.9%) |
| 65-80 | 1,109 (33.5%) | 62,190 (29.8%) |
| >80 | 230 (6.9%) | 16,675 (8.0%) |
| Female | 1,776 (53.6%) | 134,222 (64.2%) |
| Ever ICU Status | 1,063 (32.1%) | 36,823 (17.6%) |
| ALaRMS Score |  |  |
| Median [Q1, Q3] | 40.0 [30.0, 54.0] | 32.0 [20.0, 42.0] |
| Payor Class |  |  |
| Medicaid | 292 (8.8%) | 26,746 (12.8%) |
| Medicare | 1,748 (52.8%) | 88,944 (42.6%) |
| Other | 114 (3.4%) | 7,732 (3.7%) |
| Private | 1,077 (32.5%) | 79,576 (38.1%) |
| Uninsured | 81 (2.4%) | 5,927 (2.8%) |
| Staffed Bed Size |  |  |
| <100 | 110 (3.3%) | 11,023 (5.3%) |
| 100-300 | 982 (29.6%) | 70,160 (33.6%) |
| >300 | 2,220 (67.0%) | 127,742 (61.1%) |
| Teaching | 2,375 (71.7%) | 133,769 (64.0%) |
| Urban | 2,743 (82.8%) | 167,844 (80.3%) |

Abbreviations: SSI, surgical site infection; NHSN, National Healthcare Safety Network; PATOS, [infection] present at time of surgery; ICU, intensive care unit; ALaRM Score, acute laboratory risk of mortality score; SD, standard deviation

* Total may not equal 100% due to having multiple procedures in the same admission

**Supplemental Table 5. Attributable financial and clinical burden of ICD-coded SSI in admissions with procedures under NHSN surveillance for SSI, BD Insights Research Database, October 2015 through June 2019.**

|  | **Admissions with SSI N=3,312** | **Admissions without SSI, N=208,925** | **Estimated Difference/RR** |
| --- | --- | --- | --- |
| **Financial outcomes** | | | |
| Estimated total cost (95% CI) | | | |
| Unadjusted | $27,235 ($24,682, $30,051) | $16,577 ($15,070, $18,236) | $10,657 ($10,610, $10,703) ^***^ |
| Adjusted | $22,095 ($20,278, $24,076) | $15,092 ($13,885, $16,405) | $7,002 ($6,969, $7,036) ^***^ |
| **Estimated LOS (95% CI)** | | | |
| Unadjusted | 10.4 (9.7, 11.0) | 4.4 (4.1, 4.6) | 6.01 (6.0, 6.02) ^***^ |
| Adjusted | 9.0 (8.6, 9.4) | 4.1 (4, 4.3) | 4.9 (4.89, 4.91) ^***^ |
| **Clinical outcomes** | | | |
| Estimated 30-day Readmission rate (95% CI) | | | |
| Unadjusted | 14.37 (12.96, 15.92) | 5 (4.63, 5.39) | 3.19 (2.91, 3.5) ^***^ |
| Adjusted | 10.17 (9.09, 11.37) | 3.92 (3.64, 4.23) | 2.77 (2.51, 3.06) ^***^ |
| Estimated In-hospital mortality (95% CI) | | | |
| Unadjusted | 2.19 (1.7, 2.83) | 0.88 (0.73, 1.06) | 2.52 (2.09, 3.04) ^***^ |
| Adjusted | 0.64 (0.48, 0.85) | 0.42 (0.35, 0.51) | 1.53 (1.24, 1.9) ^***^ |
| Estimated HOB rate (95% CI) | | | |
| Unadjusted | 2.13 (1.65, 2.75) | 0.34 (0.28, 0.42) | 6.33 (5.23, 7.67) ^***^ |
| Adjusted | 1.41 (1.06, 1.89) | 0.24 (0.2, 0.3) | 5.88 (4.77, 7.24) ^***^ |
| Estimated COB rate (95% CI) | | | |
| Unadjusted | 0.99 (0.7, 1.38) | 0.33 (0.28, 0.38) | 3.01 (2.2, 4.12) *** |
| Adjusted | 0.42 (0.29, 0.63) | 0.21 (0.17, 0.27) | 1.98 (1.43, 2.74) *** |

Note: * <.05, ***<.0001.

Abbreviations: ICD, international classification of disease; NHSN, National Healthcare Safety Network; SSI, surgical site infection; RR, relative risk; CI, confidence interval; HOB, hospital-onset bacteremia; COB, community-onset bacteremia

Models were adjusted for age, sex, ALaRMS score, insurance payer type, and hospital-level variables (staffed bed size, teaching status, and urbanicity).

**Supplemental Table 6. Attributable financial and clinical burden of HOB in admissions with ICD-coded SSI and procedures under NHSN surveillance for SSI, BD Insights Research Database, October 2015 through June 2019.**

|  | **ICD-coded SSI with HOB N=121** | **ICD-coded SSI without HOB N=3,191** | **Estimated Difference/RR (95% CI)** |
| --- | --- | --- | --- |
|  |  |  |  |
| **Financial outcomes** | | | |
| Estimated total cost (95% CI) | | | |
| Adjusted | $54,322 ($44,367, $66,511) | $24,455 ($21,121, $28,315) | $29,868 ($28,866, $30,869) ^***^ |
| Estimated LOS (95% CI) | | | |
| Unadjusted | 27.9 (25.6, 30.5) | 9.8 (9.1, 10.7) | 18.1 (17.9, 18.3) ^***^ |
| Adjusted | 18.6 (17, 20.3) | 10.1 (9.3, 11.0) | 8.5 (8.3, 8.6) ^***^ |
| **Clinical outcomes** | | | |
| Estimated 30-day Readmission rate (95% CI) | | | |
| Unadjusted | 17.07 (11.31, 24.92) | 16.28 (14.78, 17.91) | 1.06 (0.65, 1.71) |
| Adjusted | 13.64 (8.3, 21.6) | 14.65 (11.49, 18.51) | 0.92 (0.56, 1.52) |
| Estimated In-hospital Mortality (95% CI) | | |  |
| Unadjusted | 16.42 (9.99, 25.81) | 2.07 (1.4, 3.05) | 9.29 (5.54, 15.58) ^***^ |
| Adjusted | Too low to accurately calculate | | |

Note: * <.05, ***<.0001.

Abbreviations: HOB, hospital-onset bacteremia; ICD, international classification of disease; NHSN, National Healthcare Safety Network; SSI, surgical site infection; RR, relative risk; CI, confidence interval

Models were adjusted for age, sex, ALaRMS score, insurance payer type, and hospital-level variables (staffed bed size, teaching status, and urbanicity).

**Supplemental Table 7. Model estimated financial and clinical outcomes by hospital reported SSI with HOB compared to SSI without HOB (including PATOS), October 2015 through June 2019.**

|  | **SSI admission with HOB** | **SSI admission without HOB** | **Estimated Difference/RR** | ***P*** |  |
| --- | --- | --- | --- | --- | --- |
|  | **(N=30)** | **(N=212)** | **(95% CI)** |  |  |
| **Financial outcomes** | | | | |  |
| Total cost, $ (95% CI) | $62,486  ($40,682, $95,975) | $37,900  ($26,877, $53,445) | $24,586  ($19,609, $29,563) | 0.001 | |
| LOS, days (95% CI) | 21.7 (18.4, 25.6) | 15.3 (13.2, 17.8) | 6.3 (5.7, 7.0) | <0.0001 | |
| **Clinical outcomes** | | | | |  |
| 30-day Readmission, % (95% CI) | 0.37 (0, 100) | 0.14 (0, 100) | 2.7 (0.9, 8.09) | 0.08 | |
| In-hospital mortality, % (95% CI) | 0 (0, 100) | 0 (0, 100) | 3.4 (1.06, 10.82) | 0.04 |  |

Note: The analysis did not implement a propensity-match cohort due to limited sample size.

Abbreviations: SSI, surgical site infection; HOB, hospital-onset bacteremia; PATOS, [infection] present at time of surgery; RR, relative risk; CI, confidence interval; LOS, length of stay; COB, community-onset bacteremia.

Models were adjusted for age, sex, ALaRMS, insurance payer type, and hospital-level variables (staffed bed size, teaching status, and urbanicity).
